# Topics of Public Interest

**Published:** 1915-06

**Authors:** 


### TOPICS OF PUBLIC INTEREST.

**A Just Judge.** Physicians are interested in automobile cases, both because they use that vehicle to a larger extent than any other profession except presidents and because they are citizens. The average court case tends to give the impression that the law is applied to automobilists without discretion and without regard to the legal principle of reasonable doubt and with the principle (?) "He's rich, put it on heavy." Judge Keeler of Buffalo has recently made two notable decisions. In one, the driver was charged with driving on the wrong side of the street. He claimed that the pavement was in bad condition. The Judge suspended sentence, made a personal inspection and decided that the city could not justly compel a driver to keep to any side of that street. In the second case, a man was arrested for passing a standing street car, after passengers had been discharged and while it was waiting for a cross line car. The Judge decided that the ordinance was for the protection of passengers and did not apply to a street car standing because of a delay for other reasons.

**A Clash of Idols.** Surgeon-General C. W. Gorgas, in an address in St. Louis, April 30, at the dedication of Washington University Medical School, stated that, at the end of four years work in the Isthmus, ending 1908, the malarial incidence had been reduced from 821: 1000 to 282: 1000. In 1908, all the power was concentrated in the hands of one man who made radical changes in the methods of sanitation, in spite of (then) Col. Gorgas' protests.

**Small Pox in Jamestown.** Shortly after an operation for appendicitis (findings not stated) a woman patient in the Jones General Hospital, developed small pox. The entire floor of the hospital has been quarantined.

**Bedlam,** a corruption of Bethlehem, was chartered by Henry III of England in 1247. It was a priory, near Bishop's Gate just outside the old city of London, partly prison, partly alms house, with many drunken and insane patients, whence the modern use of the name. It was a branch of the Monastery of St. Mary of Bethlehem, Palestine. In 1676, the hospital was moved to a new building on the city moat at the edge of Moorfields. In 1815, it was again moved to its present situation in St. James' Fields. (Boston M. & S. Jour.)

**Anti-Dispensing Legislation** is demanded by the Journal of the N. A. R. D. and a bill has been introduced into the Connecticut legislature, which prohibits dispensing by physicians unless they also have pharmacists' licenses. Our attitude is that the medical license, under present conditions and those of the near future, should cover all functions of the healing art. Personally, we have neither desired or considered it right to invade any special field of medicine for which the physician is not prepared and which he does not essay to cover. This statement applies both to specialization inside of the medical profession and to allied professions, such as dentistry and pharmacy. If the anti-dispensing law is passed, it should carry with it the assurance that pharmacists will carry approved preparations, with reasonable and perhaps unreasonable allowance for choice of special brands of raw materials and prepared products, by the physician; that pharmacies shall be established and kept open so as to provide for the prompt filling of prescriptions at any time and any place to which the law applies; that, in the case of the poor, the same proportionate charge for medicines shall be made as by the dispensing physician with a poor clientele, under present conditions. None of these qualifications are at present feasible and any attempt at the necessary exemptions would result in hopeless confusion. The pharmacal profession should clearly recognize one fact: the falling off of prescriptions is not so much due to office dispensing by physicians as to the reduction in the use of drugs and the relative increase of drugs administered locally, by applicators, sprays, through tubes and specula, and by hypodermatic and similar methods.

---

**Negative Results of Harrison Law.** Wm. D. McNally, Coroner's Chemist for Chicago, states that in March, the first month after the Harrison law became operative, 11 deaths from morphine occurred and 7 from the sudden withdrawal of morphine from habitues.

---

**Oysters a Safe Food.** Survey of oyster beds by the Public Health Service and Dept. of Agriculture, in co-operation with state authorities and with the incentive to caution from purely business reasons, has rendered pollution by sewage bacteria practically nil, from Massachusetts to Virginia. The control of the packing and shipping of oysters has greatly diminished the practices of "floating" and "fattening" by imbibition of fresh water.

(Note: It should not be forgotten that, however safe oysters may be, they are of very little food value. Barring a slight and variable amount of glycogen, they contain about 5 percent. of protein so that a kilogram of oyster meat represents only the minimum protein ration and scarcely 10 per cent. of the total ration in calories.)

**Examinations for Assistant Surgeon, U. S. Public Health Service** will be held June 21 at various places in the country. One year's hospital or two professional experience; 23 to 32 years of age; 5 feet 4 in. to 6 feet 2 in., height; citizenship, medical diploma, are the principal requirements. Initial salary $2,000. Candidates should apply immediately to the Surgeon-General at Washington.

---

**$10,000,000** is to be spent for a medical school, including research buildings, and hospital by Columbia, in connection with the Presbyterian Hospital. The University will provide three-fourths of the fund and five years are allowed for raising the total sum. Our magnificent philanthropies are a considerable factor in the increased cost of living. We question whether such a scale of expenditure affords quite the appropriate atmosphere for preparing young men for the meagre receipts and close economies of medical practice.

---

**Military Camp** at Niagara-on-the-Lake. In view of the increased attendance at this encampment this year, special precautions will be taken to protect the water supply. A mechanic filtration plant will be established, under the direction of Lt. G. J. Fitzgerald, Asst. Prof. of Hygiene of Toronto University both for the camp supply and for the village. 800 Toronto and McGill students took a ten days' officers' training course during May.

---

**Iola Sanitarium.** The corner stone of the new service building was laid May 1. The cost of the addition will be $150,000.00. 90 more patients will be accommodated. This institution is the Monroe Co. Tuberculosis Hospital and is a model of similar institutions. Dr. John F. W. Whitbeck is President of the Board of Managers. Dr. Montgomery E. Leary, recently elected Vice-president of the Medical Society of the State of N. Y. is Superintendent.

---

**Multiple Birth—Fake Report.** A case of simultaneous birth of 3 boys and 5 girls, circumstantially reported in the Boston Medical and Surgical Journal of Sept. 26, 1872 and quoted by Gould and Pyle in their Anomalies and Curiosities of Medicine, has been shown to be a pure fake.

---

**15 Medical Missionaries are Needed** by the American Board of Commissioners for Foreign Missions; 9 for China, 4 for Turkey, 1 for Africa, 1 for relief work at Monastir, Serbia. A medical degree, interne service, "earnest Christian consecration" but no sectarian test, aged under 35, are the requirements. Address Dr. C. H. Patton, 14 Beacon St., Boston. The board anounces the death from typhus of a Swiss nurse at Sivas, Turkey; also the conferring on Dr. Hardman N. Kinnear of the Chinese decoration of the Red Cross with life membership in the society, for services to the wounded near Foochow in 1911.

---

**State Board Statistics** (Jour. of the A. M. A.). Of 3594 medical graduates in 1914, only 2868 came before state boards in the current year and only 2504 passed. Many of the remaining will undoubtedly apply for licenses in 1915 but of all graduates of 1910-14 examined in 1914, there were only 4549, of whom 3748 passes. As this includes re-examinations, it appears that many graduates fail to secure licenses. The total number of examinations for the year was 5570. 1381 physicians went from one state to another for licenses in 1914, indicating a movement of more than 1% per annum of the medical population.

---

**The Thompson Bill** requiring detailed reports of receipts and expenditures by charitable organizations, has been vetoed by Gov. Whitman.

---

**The Bill Increasing the Automobile Tax** has also been vetoed.

---

**The Niagara Falls Memorial Hospital** held its fifteenth annual commencement exercises May 11. The following nurses received diplomas: Miss Myrtle Brand, Princeton, Ont.; Miss Nell D. Shepard, Barrie, Ont.; Miss Vera Wallace, Merritton, Ont.; Miss Hattie Wright, Toronto, Ont.; Miss Emma Edbrook, this city; Miss Charlotte Crompton, New York; Miss Eva Wood, this city; Miss Mary Graves, Lima; Miss Mary Metcalf, Burford, Ont.; Miss Ethel Stoffer, this city; Miss Kathleen Southcott, Saint Catharines, Ont.; Miss Ethel Robinson and Miss Lydia Gray, Niagara Falls, Ont.

---

**National Prohibition.** Oscar W. Underwood, in the House of Representatives, has made a strong argument against this movement. Financially, it would cost the government nearly $735,000,000 a year in loss of customs, excise, income taxes, etc. It would virtually confiscate $771,000,000 worth of capital and would put about 77,000 men out of employment. A comparison of prohibition with other states shows no favorable sociologic condition, rather the contrary. Finally, he considers the measure an encroachment on the rights of states —not to mention individuals—and states that any legislation not supported by general sentiment is a farce. We have previously expressed our opinion on legislation of this kind. By heredity and personal habit, the writer represents a century and a third of total abstinence and believes that alcohol has no place as a beverage. But he feels that at present, the greatest danger of this country is not any particular wrong but the invasion of personal liberty in regard to which, we have gone farther than any other government of the world that makes any claims to freedom of the individual.

---

**Animal Hospital.** The Buffalo Society for the Prevention of Cruelty to Animals is planning a hospital for the care of sick and injured domestic animals, somewhat on the lines of the Angell Memorial Hospital of Boston, recently opened. Contrary to a rather prevalent idea, we believe that the majority of the medical profession are heartily in favor of such a hospital, both as a matter of mercy and because such an institution conducted in a proper spirit of co-operation, will tend in many ways to advance knowledge along lines of scientific medical interest and susceptible of practical deductions for the treatment of human patients.

---

**A U. S. Civil Service Examination** will be held for the appointment of a (male) **Physiologist** in the Bureau of Animal Industry. The salary is $2500, increasing to $3000. Successful applicants, if not appointed to the present vacancy, will be eligible to similar openings as they may occur. The degree of M. D. or Ph. D. and four years' experience in physiologic work are required. No formal examination will be held but applicants will be rated up to 40 points on education, up to 50 on experience and up to 10 on publications. Write for forms 304 and 2095, stating position sought, addressing U. S. Civil Service Commission, Washington. Blanks must be returned by June 8. As notices of this Commission are usually received either too late for publication or so as to allow for very meagre time, we would state that applicants may apply at the New York Custom House.

---

**Contributions for Helping Homeless Children,** on the Russian Frontier, may be sent to H. S. H. Prince Hatzfeldt, German Embassy, Washington. The appeal is made by four noble and two untitled American-born ladies.

---

**Contributions to the Fund for the Relief of Belgian Physicians,** may be sent to American Medicine, N. Y. We understand that instruments and French medical books can be used.

---

**Old Linen, Knit Goods, etc.,** are needed for the preparation of dressings for the wounded in Europe, also help in their preparation, not necessarily requiring medical or nursing education. Offers should be made to the War Relief Committee, 181 Franklin St., Buffalo.

---

**Contributions for the Serbian Relief Fund** may be sent to J. P. Morgan & Co., marked "Serbian Sanitary Relief."

---

**Colorado** has passed an amended medical law, providing for the licensing of osteopaths, chiropractors, etc., with lower standards for drugless healing.

---

**Napropathy** founded by Oakley Smith, as a result of his appreciation of the inadequacy of regular medical science after two years undergraduate study at the Iowa University College of Medicine, is represented by a college and journal. Like medicine, it has a peculiar vocabulary, e.g., Ligatight, a tight ligament; Symptora, a functional or organic disturbance due to disease located somewhere else, as pneumonia due to a diseased spinal ligament, Bright's disease due to trouble with the lower dorsal ligaments; Vertebond, the ligaments and fasciae joining two vertebrae. Dr. Cuthbert Powell, Col. Med., May, 1915.

---

**Pay for Birth and Death Certificates.** The Seely bill, signed by Gov. Whitman, provides for a 25 cent fee to be paid by municipalities to physicians for certificates properly filed.

---

**The Question of Legalizing Abortion in Cases of Military Rape** is being seriously discussed, in Europe. Dr. Depasse, Bull. et Mem. de la Soc. de Med. de Paris, Mch. 1915, mentions a number of cases, 6 then under observation by Dr. Cabanes. A particularly sad case was that of a mother and two daughters, raped in their own home by 17 German soldiers, all pregnant. In various instances, pregnant women had threatened suicide if not aborted and he knew of two instances in which the threat had been carried out. German physicians have reported practically identical experiences and have raised the same ethical and legal issues. It is a sad old story, repeated in every war and with little regard for nationality though, of course, less frequent under modern and more enlightened military rules and discipline.

---

**Duty of Medical Society to Defend Member.** The Minnesota Supreme Court, Penhall vs. State Medical Assn. has ruled that the fact that the claims of a mal-practice suit developed before the passage of by-laws providing for defense of members, does not relieve the society of its responsibility, although conceding that if the physician sued had not been a member of the society at the time, such responsibility would not have existed.

---

**The Volta Bureau** for the Increase and Diffusion of Knowledge Relating to the Deaf, will send literature to any one interested. This deals with training, not medical treatment. Address at 1601 35th St., N. W., Washington.

---

**No Exemption From Speed Laws for Physicians.** The Mayor of St. Louis has vetoed an ordinance exempting physicians from the operation of speed laws, but suggests a distinguishing mark for physicians' autos and discretionary powers on the part of the police to allow physicians to pass through processions, etc. This is sensible. Haste makes waste, even of time. We have heard of medical officials without emergency practice or duties, deliberately taking advantage of their political influence to secure special privileges of this nature and have observed exemption from ordinary police regulations, not on account of any official duty or humanitarian reason, but simply because the official wanted to see a procession. For instance, a fire department, passenger auto., deliberately passing to the left around the McKinley monument, without any fire. In this country, no office-holder should be above the law and should be allowed to violate it only as to technicalities, when necessary in the direct discharge of duties. And there should be no privileged class except for very urgent reasons of an analogous nature.

---

**Brevium**, a radio-active disintegration product of uranium, has been announced as a newly discovered element, by Prof. Goehring of Karlsruhe.

---

**The Warren Triennial Prize** of $500 will be awarded on essays received up to April 14, 1916, under the usual conditions of secrecy. Essays must be type-written, in English, French or German, suitably bound, and must represent original, unpublished work in physiology, surgery or pathologic anatomy. Address Dr. Frederic A. Washburn, Resident Physician, Massachusetts General Hospital, Boston.

---

**Harrison Law Ruling.** A physician visiting a patient at a hospital is subject to the same exemption as at a private house but the hospital must preserve for two years, a record of date, amount and drug dispensed. The physician must sign his initials to the chart or medicine sheet. National government, state, county or municipal hospitals, established and maintained solely as such, are further exempt.

